# Bronchopulmonary Dysplasia: A Five-Year Retrospective Cohort Study on Differences in Clinical Characteristics and Morbidities According to Severity Grading

**DOI:** 10.7759/cureus.42720

**Published:** 2023-07-31

**Authors:** Estela Kakoo Brioso, Joana Moscoso, Duarte Malveiro, Marta Aguiar, Madalena Tuna

**Affiliations:** 1 Pediatrics, Centro Hospitalar Lisboa Ocidental, Lisbon, PRT; 2 Pediatrics, Hospital De Cascais Dr. José De Almeida, Lisbon, PRT; 3 Comprehensive Health Research Center, Nova Medical School, Lisbon, PRT

**Keywords:** periventricular leukomalacia, germinal matrix hemorrhage and intraventricular hemorrhage, retinopathy of prematurity, sepsis, necrotizing enterocolitis, bronchopulmonary dysplasia

## Abstract

Introduction: Bronchopulmonary dysplasia (BPD) is the most common complication associated with extreme prematurity. Although several criteria defining severity were developed over time, there are a few studies describing the differences in BPD phenotype and neonatal morbidities and complications between severity groups. We aimed to describe these differences in BPD patients of a neonatal intensive care unit (NICU).

Methods: We conducted an observational retrospective cohort study through a medical record review over a five-year period. Participants were newborns admitted to an NICU who were diagnosed with BPD. We performed a descriptive statistical analysis of gestational complications and the use of antenatal corticosteroid therapy, birth-related data, and complications throughout the NICU stay, as well as the respiratory support used. We also compared different severity groups across these variables. The patients were divided into severe and non-severe BPD using the severity criteria of the 2001 NICHD/NHLBI/ORD consensus workshop.

Results: A total of 101 newborns with BPD participated in the study and 73 had data on BPD severity. The median gestational age was 27 weeks, ranging from 23 to 32 weeks. Of these 73 newborns, 36 had mild BPD (49.3%), 10 had moderate BPD (13.7%), and 27 had severe BPD (37.0%). When comparing severe and non-severe BPD, we found that extreme prematurity, extremely low birth weight, and small size for gestational age were more frequent in the severe BPD group (*p*-value=0.012, *p*-value<0.001, and *p*-value=0.012, respectively). Infants with severe BPD had a longer duration of invasive ventilation than those with mild or moderate BPD (*p*-value<0.001). Late sepsis, necrotizing enterocolitis, severe brain injury, and retinopathy of prematurity were more frequent in severe BPD (*p*-value=0.017, *p*-value=0.045, *p*-value=0.033, *p*-value=0.003, respectively).

Discussion: Previously published evidence describing causal links between BPD development and comorbidities exists but data on their impact on BPD severity are scarce. In our study, severe BPD seemed to be associated with a higher frequency of comorbidities and complications. Further studies are needed to ascertain the impact of each morbidity on the severity of BPD and if measures to prevent them could lead to potentially milder BPD disease.

## Introduction

Premature birth affected an estimate of 8.7% to 13.4% of all live births in the world, in 2014 [1]. Bronchopulmonary dysplasia (BPD) is the most common complication associated with extreme prematurity, affecting 47% of all extremely-low-gestational-age newborns (ranging from 24% of those born at 28 weeks of gestational age to 100% of those born at 22 weeks) [2,3]. BPD prevalence is increasing, most likely due to the increased survival of extremely-low-gestational-age newborns [2-4]. In fact, prematurity and low birth weight are the strongest risk factors for BPD [3,5]. Respiratory support, male sex, pulmonary hypertension, and intrauterine growth restriction are also associated with a higher risk of developing BPD [5-7]. Other risk factors with a possible contribution to pulmonary deterioration include late surfactant deficiency [8], patent ductus arteriosus (PDA) [9,10], and sepsis [3,11].

Over the years, advancements in perinatal medicine such as antenatal corticosteroids and exogenous surfactant therapy have significantly reduced neonatal morbidity and mortality [2,4]. Interventions with benefits in reducing the risk of BPD include treatment with caffeine in extremely preterm infants [12], avoidance of intubation and ventilation in the first minutes of life with preference for the use of non-invasive respiratory support [13], use of low tidal volumes to minimize volutrauma [14], defining oxygen saturation targets of 91-95% [15], administration of vitamin A [16], and postnatal corticosteroids [3,17,18]. These strategies led to a decline in BPD in less premature infants, giving way to a “new BPD”: a milder form of respiratory disease in extremely-low-gestational-age newborns [4]. As such, nowadays, premature infants with BPD have characteristically less emphysema, fibrosis, and airway changes [4]. They also present lung alterations resulting from an arrest of lung development at the late canalicular or early saccular stage with subsequent alveolar simplification with fewer and dysmorphic capillaries due to various antenatal and postnatal insults [4]. Despite these therapeutic advancements, BPD remains associated with neurodevelopmental impairment and growth failure [19,20].

From 2001 to 2017, BPD was defined as a requirement for supplemental oxygen for at least 28 days at a postmenstrual age of 36 weeks (or at the time of discharge to home, if earlier) [21]. This definition resulted from the 2001 National Institute of Child Health and Human Development (NICHD)/National Heart, Lung and Blood Institute (NHLBI)/Office of Rare Diseases (ORD) workshop organized to review previous definitions and define a consensus that would better characterize the “new BPD.” In 2018, a new NICHD consensus workshop further refined these diagnostic criteria, including respiratory death before the 36th week of postmenstrual age as a criterion and defining new severity grades [22]. Although the 2018 criteria categorized these patients better according to modern respiratory care, they were still based solely on expert consensus and lacked appropriate prognostic discrimination between groups. In 2019, data-driven new diagnostic criteria were published defining BPD in very preterm infants as need of any respiratory support at 36 weeks of postmenstrual age, with no need for assessment of supplemental oxygen use prior to 36 weeks of postmenstrual age [23]. BPD severity was graded according to the type of respiratory support at 36 weeks of postmenstrual age: nasal cannula ≤2 L/min (grade I), nasal cannula >2 L/min or noninvasive positive pressure ventilation (grade II), or invasive positive pressure ventilation (grade III) [23]. These new criteria predicted early childhood mortality and morbidity (respiratory and neurodevelopmental) better than previous published criteria [23].

Despite the several criteria developed over time, there are a few studies describing the differences in BPD phenotype and risk factors between severity groups and, specifically, differences in neonatal morbidities and complications. As such, we aimed to describe these differences in a retrospective cohort of BPD patients in a neonatal intensive care unit (NICU).

## Materials and methods

Study design

We conducted a retrospective cohort study through a medical record review.

Inclusion and exclusion criteria

Participants in this study were all the newborns admitted to the NICU of a level 3 hospital between January 1st, 2015, and December 31st, 2019 (five years), who were diagnosed with BPD. BPD was diagnosed using the 2001 NICHD/NHLBI/ORD consensus workshop criteria. No participants were excluded from this study.

Data collection

We collected relevant data from the electronic medical record of each participant on the peripartum period, as well as the gestational period, and from admission into the NICU until discharge (or transfer) or death.

Birth weight lower than 1000 g was defined as extremely low; birth weight between 1000 and 1499 g as very low; and birth weight between 1500 and 2499 g was defined as low. Size for gestational age was defined as follows: birth weight below the 10th percentile was classified as small (SGA), birth weight between the 10th and 90th percentile was classified as appropriate (AGA), and birth weight above the 90th percentile as large (LGA). Respiratory distress syndrome was categorized according to radiological criteria: Grade I for mild diffuse reticulogranular opacities, grade II for ground-glass opacities and air bronchogram, grade III for generalized air bronchogram and poorly defined cardiac silhouette, and grade IV for generalized loss of lung transparency and loss of cardiac silhouette. We acknowledged the diagnosis of sepsis in any infant manifesting systemic signs of infection for whom antibiotics were administered. Sepsis was classified as early or late depending on whether the onset of symptoms was in the first 72 hours of life or afterward, respectively. Severe brain injury was defined as having grade III or IV germinal matrix hemorrhage and intraventricular hemorrhage (GMH-IVH) or grade II or higher periventricular leukomalacia (PVL). No data on the use of vitamin A were collected since no formulation for intramuscular use was available in Portugal at the time of this study. Also, no data on caffeine usage were used as it is routinely administered to all infants with less than 34 weeks of gestational age.

Statistical analysis

Statistical analysis was done using the IBM® SPSS® Statistics version 22 software (IBM Corp., Armonk, NY). We performed a descriptive statistical analysis of gestational complications and the use of antenatal corticosteroid therapy, birth-related data (including the presence of respiratory distress syndrome and surfactant administration), and complications throughout the NICU stay (sepsis, necrotizing enterocolitis [NEC], hemodynamically significant PDA, retinopathy of prematurity [ROP], GMH-IVH, and PVL), as well as the respiratory support used. We also compared different severity groups across these variables. For this, the patients were divided into severe and non-severe (mild or moderate) BPD using the severity criteria of the 2001 NICHD/NHLBI/ORD consensus workshop. When the assumptions for parametric tests were met, we used the t-test for comparing continuous variables and the chi-square test for categorical variables. Otherwise, we used the Mann-Whitney U and Fisher tests, respectively. Statistical significance was set at *p*-values ≤0.05.

## Results

During the study period, 1218 newborns were admitted to the NICU. One hundred and one newborns were diagnosed with BPD and were thus included in the study. Most newborns had extremely low birth weight (n=63; 62.4%), 34 had a very low birth weight (33.7%), and only four had a low birth weight (4.0%) (Table 1). Median birth weight was 896 g (birth weight was between 408 and 1634 g). Nonetheless, most were AGA (80.2%) and only 16.8% were SGA. The median gestational age was 27 weeks; 23 weeks was the lowest gestational age found and 32 weeks the highest. Fifty-five newborns were male (54.5%).

**Table 1:** Participants' data *n=97 (four newborns lacked ophthalmology observation). Continuous variables are represented as median (P25-P75) and categorical variables as n (%). AGA: Appropriate for Gestational Age; GMH-IVH: Germinal Matrix Hemorrhage and Intraventricular Hemorrhage; HFNC: High-Flow Nasal Cannula; IMV: Invasive Mechanical Ventilation; LGA: Large for Gestational Age; nCPAP: Nasal Continuous Positive Airway Pressure; PDA: Patent Ductus Arteriosus; PVL: Periventricular Leukomalacia; RDS: Respiratory Distress Syndrome; SGA: Small for Gestational Age.

Participants' data (n=101)			
Newborn data			
Gestational age (weeks)	27 (25-29)	Size for gestational age	
Birth weight (g)	896 (698-1094)	- SGA	17 (16.8)
Low birth weight	4 (4.0)	- AGA	81 (80.2)
Very low birth weight	34 (33.7)	- LGA	3 (3.0)
Extremely low birth weight	63 (62.4)	Male gender	55 (54.5)
Gestational complications			
Gestational diabetes	7 (6.9)	Early pregnancy bleeding	15 (14.9)
Hypertension	19 (18.8)	Urinary tract infection	5 (5.0)
Fetal growth restriction	23 (22.8)	Incompetent cervix	8 (7.9)
Premature rupture of membranes	25 (24.8)	Oligohydramnios	8 (7.9)
Chorioamnionitis	13 (12.9)	Polyhydramnios	1 (1.0)
Labor/delivery			
Spontaneous labor	45 (44.6)	Vaginal delivery	18 (17.8)
Labor induced due to maternal illness	14 (13.9)	Cesarean delivery	83 (82.2)
Labor induced due to fetal illness	42 (41.6)		
Antenatal corticosteroid therapy for fetal maturation			
None	5 (5.0)	Complete	73 (72.3)
Partial	23 (22.8)		
Respiratory distress syndrome (hyaline membrane disease)			
Grade I	17 (16.8)	Grade III	24 (23.8)
Grade II	51 (50.5)	Grade IV	9 (8.9)
Respiratory support			
Surfactant administration	79 (78.2)	Need of IMV	89 (88.1)
Need of nCPAP or HFNC	95 (94.1)	Duration of IMV (days)	16 (6-40.5)
Complications			
Early sepsis	82 (81.2)	Necrotizing enterocolitis	13 (12.9)
Late sepsis	75 (74.3)	- Surgical enterocolitis	5 (38.5)
Hemodynamically significant PDA	30 (29.7)	Severe brain injury	26 (25.7)
- Pharmacologic therapy	28 (93.3)	GMH-IVH	59 (58.4)
- Surgical closure	17 (56.7)	- Grade I	27 (45.8)
Retinopathy of prematurity*	43 (44.3)	- Grade II	16 (27.1)
- Stage 1	8 (18.6)	- Grade III	16 (27.1)
- Stage 2	18 (41.9)	- PVHI	0 (0.0)
- Stage 3	17 (39.5)	PVL	50 (49.5)
- Stage 4 or 5	0 (0.0)	- Grade 1	34 (68.0)
- Plus disease	8 (18.6)	- Grade 2	12 (24.0)
- Submitted to surgery	12 (27.9)	- Grade 3	2 (4.0)
		- Grade 4	2 (4.0)
Mortality	7 (6.9)		

Prematurity was spontaneous in 45 newborns (44.6%), induced due to fetal illness in 42 cases (41.6%), and 14 due to maternal illness (13.9%). Cesarean was the most frequent mode of delivery (82.2%). Antenatal corticosteroids were given for fetal maturation in 95.0% and, in 73 (72.3%), it was possible to administer all the corticosteroid doses before delivery.

The most frequent gestational complications were premature rupture of membranes (n=25; 24.8%), fetal growth restriction (n=23; 22.8%), hypertension (n=19; 18.8%), early pregnancy bleeding (n=15; 14.9%), and chorioamnionitis (n=13; 12.9%).

Most newborns required invasive mechanical ventilation (n=89; 88.1%); the median duration of ventilation was 16 days and the maximum was 100 days. Surfactant was administered in 79 newborns (78.2%). Almost all BPD newborns (n=95; 94.1%) needed noninvasive positive airway pressure, either as a first respiratory support modality or for ventilator weaning.

During the stay at the NICU, 82 (81.2%) newborns were diagnosed with early sepsis and 75 with late sepsis (74.3%). Thirty newborns had a hemodynamically significant PDA (29.7%); most underwent pharmacologic therapy (n=28; 93.3%) and 17 needed surgical closure (56.7%). NEC was found in 13 newborns (12.9%) and five underwent surgical treatment (38.5%). Neurologic complications were also found in these newborns: 59 had intraventricular hemorrhage (58.4%) and 16 had grade II or higher PVL (15.8%). Four infants had no data on the ophthalmology exam. Of the 97 remaining, 43 newborns were diagnosed with ROP and 12 were submitted to surgery.

The mortality rate among newborns with BPD in this study was 6.9% (n=7). At birth, these newborns had a median weight of 650 g (minimum of 475 g) and a median gestational age of 26 weeks (minimum of 23 weeks). The median age at death was 98 days.

We were able to collect data on BPD severity grading in 73 newborns (the remaining were transferred to other hospitals due to surgery need or for continued care nearer to home). Of these 73 newborns, 36 had mild BPD (49.3%), 10 had moderate BPD (13.7%), and 27 had severe BPD (37.0%) (Table 2). When comparing severe and non-severe BPD, we found that extreme prematurity (<28 weeks), extremely low birth weight, and SGA size were more frequent in the severe BPD group (*p*-value=0.012, *p*-value<0.001, and *p*-value=0.012, respectively). No difference was found concerning the complete administration of antenatal corticosteroids (*p*-value=0.222). Infants with severe BPD had a longer duration of invasive ventilation (median 42.5 days) than those with mild or moderate BPD (median 7.5 days) (*p*-value<0.001).

**Table 2:** Comparison of antenatal and infant characteristics between infants with severe and non-severe BPD *n=69 (four newborns lacked ophthalmology observation). Continuous variables are represented as median (P25-P75) and categorical variables as n (%). When the assumptions for parametric tests were met, we used the t-test for comparing continuous variables and the chi-square test for categorical variables. Otherwise, we used the Mann-Whitney U and Fisher tests, respectively. ACTFM: Antenatal Corticosteroid Therapy for Fetal Maturation; IMV: Invasive Mechanical Ventilation; PDA: Patent Ductus Arteriosus; RDS: Respiratory Distress Syndrome; ROP: Retinopathy of Prematurity.

	Bronchopulmonary dysplasia			
	Total	Non-severe	Severe	p-Value
	n=73	n=46	n=27	
Gestational age (weeks)	27 (25-29)	28 (27-29)	25 (24-27)	<0.001
Extreme preterm (<28 weeks)	43 (58.9)	22 (47.8)	21 (77.8)	0.012
Birth weight (g)	880 (698-1094)	1029 (842.5-1143.5)	689 (564-813)	<0.001
Extremely low birth weight (<1000 g)	45 (61.6)	20 (43.5)	25 (92.6)	<0.001
Small for gestational age	13 (17.8)	4 (8.7)	9 (33.3)	0.012
Male gender	41 (56.2)	29 (63.0)	12 (44.4)	0.122
Complete ACTFM	57 (78.1)	38 (82.6)	19 (70.4)	0.222
Cesarean delivery	60 (82.2)	36 (78.3)	24 (88.9)	0.348
RDS (grade III or IV)	22 (30.1)	8 (17.4)	14 (51.9)	0.002
Surfactant administration	59 (80.8)	35 (76.1)	24 (88.9)	0.180
IMV	64 (87.7)	38 (82.6)	26 (96.3)	0.141
Duration of IMV (days)	15.5 (6-38.8)	7.5 (4.00-15.75)	42.5 (20.25-57.00)	<0.001
Early sepsis	63 (86.3)	39 (84.8)	24 (88.9)	0.735
Late sepsis	53 (72.6)	29 (63.0)	24 (88.9)	0.017
Hemodynamically significant PDA	21 (28.8)	10 (21.7)	11 (40.7)	0.083
Necrotizing enterocolitis	8 (11.0)	2 (4.3)	6 (22.2)	0.045
Severe brain injury	17 (23.3)	7 (15.2)	10 (37.0)	0.033
Retinopathy of prematurity*	32 (46.4)	15 (33.3)	17 (70.8)	0.003
ROP with need of surgery	8 (11.6)	1 (2.2)	7 (29.2)	0.002

Early sepsis was equally frequent among severe and non-severe disease (*p*-value=0.735) but late sepsis was more frequent in newborns with severe BPD (*p*-value=0.017).

NEC was also more frequently present in the severe disease group (*p*-value=0.045). No difference in the frequency of hemodynamically significant PDA was found between severe and non-severe BPD (*p*-value=0.083). However, 38.9% (seven out of 18) of infants with a hemodynamically significant PDA ended up needing supplemental oxygen at discharge; only 10.4% (five out of 48) of those without a hemodynamically significant PDA needed supplemental oxygen at discharge (*p*-value=0.013).

Infants with severe BPD had a higher proportion of severe brain injury (37.0%) than infants with mild or moderate BPD (15.2%) (*p*-value=0.033). ROP was more frequently found in severe BPD infants (70.8% vs 33.3%, *p*-value=0.003) and they needed surgery for ROP more frequently (29.2% vs 2.2%; *p*-value=0.002).

## Discussion

BPD is primarily a disease of premature newborns, particularly the ones with the lowest birth weight, and, as expected, our results showed that extreme prematurity, extremely low birth weight, and being small for gestational age were associated with more severe BPD. Similar results were found in other studies [24].

Although the administration of antenatal corticosteroids for fetal maturation is important to reduce complications in the newborn, it is not always effective in preventing the development of BPD. In fact, in our study, most received antenatal corticosteroid therapy (95%), including a full-course administration in 72%. We could not find any difference in BPD severity with antenatal corticosteroid therapy, but this might be the result of a lack of statistical power due to the low number of newborns who had not received antenatal corticosteroids.

Severe respiratory failure was prominent among these newborns. Most required respiratory support with invasive mechanical ventilation, even though the surfactant was administered to 78.2%. These are not surprising results as our cohort is exclusively composed of infants with BPD and three-quarters had less than 29 weeks of gestational age. Probably for the same statistical reason as the use of antenatal corticosteroids, we could not find a difference in the surfactant use between severity groups.

The long duration of ventilation we encountered (median 15.5 days) seemed to be in association with more severe BPD. In fact, severe BPD infants had a median duration of ventilation more than five times longer than that of non-severe BPD. Although, by definition, more severe BPD is associated with higher respiratory support, this relation also reflects a more damaged lung. Besides this, the neonatal lung is immature and underdeveloped, and, therefore, more prone to ventilator-induced injury.

Perhaps in relation to risk factors for preterm labor, most of the BPD newborns were diagnosed with early sepsis. Surprisingly, late sepsis was present in almost three-quarters of the newborns and seemed to be more frequent in severe BPD patients. The inflammatory response associated with sepsis contributes to lung injury and the subsequent need for higher respiratory support and is already established as a risk factor for developing BPD [3,11]. Furthermore, severe BPD infants frequently have a longer length of stay and a longer duration of ventilation, which increases the risk of developing sepsis [10,24]. Device-related infections might have accounted for this high prevalence of late sepsis as this population frequently requires multiple procedures for comorbid conditions (such as orotracheal intubation or central venous access) and parenteral nutrition, which increase the risk of infection. It is, therefore, difficult to ascertain if sepsis is responsible for more severe BPD or if it is, in fact, the more severe BPD that predisposes infants to develop sepsis. In contrast to our results, a study that included 9939 infants with BPD found lower frequencies of both early and late sepsis (1.5% and 16.4%) [24]; however, in this study, sepsis was only diagnosed when a positive culture was found, whereas, in our study, diagnosis of sepsis was based upon suspected infection. Despite different criteria and the overall incidence, they also found a higher frequency of both early and late sepsis among infants with more severe BPD.

Despite being a respiratory condition, BPD is frequently encountered in extremely premature newborns with multiple comorbid conditions and complications affecting several organ systems. These include hemodynamically significant PDA, NEC, GMH-IVH, PVL, and ROP. PDA has long been associated with BPD, with frequencies varying between studies according to the inclusion of all PDA (72.2%) or only the hemodynamically significant PDA (10.3%) [9,24,25]. Our study showed 29.7% hemodynamically significant PDA; however, we did not find any difference in hemodynamically significant PDA between the severity groups. In fact, early routine treatment of moderate-to-large PDA does not seem to reduce BPD incidence among premature infants, as shown in the PDA-TOLERATE trial [26]. On the other hand, a more recent post-analysis of that trial showed evidence of greater severity of BPD in infants not offered early routine treatment of moderate-to-large PDA who were intubated for 10 days or longer [10]. This finding illustrates the complex relationship between PDA and BPD. To date, we have yet to understand the exact impact of PDA on BPD, particularly if the presence of PDA increases the risk of developing BPD or leads to more severe BPD [27].

NEC is more frequent in infants with risk factors for developing BPD (prematurity and low birth weight). Besides having common risk factors, there might also exist a causative role of NEC in the development of BPD, at least indirectly. A recent Spanish study found that infants with NEC had a higher risk of developing BPD [28]. Infants with NEC often develop sepsis and multiorgan failure with a frequent need for invasive mechanical support, which can induce lung injury. The inflammatory response associated with NEC might further contribute to lung injury and a subsequent need for supplemental oxygen [3].

The frequency of NEC among infants with BPD in our study (12.9%) was in line with the recent data from the United States [24]. In our study, the severity of BPD also seemed to be associated with the diagnosis of NEC. Similarly, a study of 92 infants with NEC showed an association between surgical NEC and higher BPD severity [29].

Neurologic injury is one of the most dreaded complications in premature infants. In our study, we found GMH-IVH and PVL in 58.4% and 49.5%, respectively. Severe brain injury was present in 25.7% and was more common among infants with severe BPD. Jensen et al. (2021) also observed a higher frequency of severe brain injury in grade III BPD infants than in grade I or II BPD infants (23.4% vs 13.1%) [24].

Another risk factor for neurodevelopmental impairment in premature infants is ROP, particularly when severe. In our study, 44.3% of the infants were diagnosed with ROP and 27.9% of those underwent surgery. Our results are similar to the recently published data on DBP newborns [24]. The etiology of ROP is multifactorial but, in probable relation with the higher and longer supplemental oxygen needs, we found that more severe BPD was associated with a higher frequency of ROP.

Infants with BPD require more days of invasive mechanical ventilation and have higher oxygen needs than non-BPD infants; as such, it is only natural that BPD patients have more severe forms of retinopathy [30]. Potentially for the same reasons, more severe BPD was also associated with a significantly higher incidence of ROP in our study.

Our study contributed to filling the gap in the literature regarding factors associated with BPD severity. Most published studies focus on differences between BPD and non-BPD infants but there is a paucity of studies comparing clinical characteristics of infants with BPD across different severity groups. Data comparison between published evidence is also challenging as different classifications for BPD and different severity gradings are used.

In our study, we did not compare a BPD and non-BPD sample and, thus, we are unable to state which factors are associated with both the development and severity of BPD. However, multiple studies have already been published documenting risk factors for the development of BPD.

The small sample size and the difficulty in ascertaining causality are the most important limitations of our study. It is possible that some differences between groups were not found due to a lack of statistical power. However, even with this sample size, the two severity groups had differences in several clinical factors, probably translating to strong associations. Unfortunately, the retrospective nature of our study does not allow us to firmly affirm the causality of the associations we found, specifically because prematurity and low birth weight are important confounders. It would be interesting to conduct an observational prospective study to determine how much each clinical characteristic contributes to the severity of BPD, as well as understanding the impact that more severe disease has in the development of complications.

In the future, precision medicine may play an important role in the care of BPD patients and hopefully improve outcomes. A more individualized approach to ventilation strategies using novel monitoring techniques such as electrical impedance tomography and electromyography of the diaphragm may provide additional benefits beyond the currently used ventilatory strategies [31]. Understanding the interindividual variations of the pharmacokinetics and pharmacodynamics of caffeine through pharmacogenetic studies might lead to a more precise dosing strategy and increased efficacy of caffeine [31]. Analysis of volatile organic compounds present on exhaled breaths of newborns can potentially identify individuals at higher risk of developing BPD and who could potentially benefit from earlier interventions to reduce the incidence of BPD [31].

## Conclusions

Although the risk of developing BPD is largely associated with extreme prematurity and extremely low birth weight, both nonaddressable by the neonatology care team, prevention of severe BPD must be a priority. Besides the usual care with the appropriate choice of ventilation and the use of surfactant, caffeine, and corticosteroids when appropriate, we highlight the importance of instating measures to prevent late sepsis and NEC, both morbidities associated with more severe disease with a probable role in the development of more severe BPD. Additionally, special focus on preventing severe neurological injury and ROP should be given to infants with severe BPD, as both conditions seem to be more prevalent in this group of patients and can potentially contribute to a worse neurodevelopmental prognosis and quality of life.
